# National Association of Dental Faculties

**Published:** 1896-09

**Authors:** 


### National Association of Dental Faculties.

This body held its annual meeting at Saratoga, N. Y., beginning August 1st, 1896, in the hall at the Grand Union Hotel. The attendance was large, almost every college having representation in the association had its delegate present. There was quite a number of applications for membership, four of which were reported upon favorably by the Executive Committee, and will be presented for final action at the next meeting. Several subjects came up for consideration, the discussion of which, and the final action had, clearly indicated marked progress. The importance of better preliminary attainments on the part of students generally, elicited much discussion, the drift of which indicated clearly the growing desire for advancement in this respect. There was also manifested a desire to extend the curriculum. The extension of the time of the course, which met with considerable opposition two or three years ago, had now, seemingly, the acquiescence of all, and the same may be said in regard to the extension of time for graduation from two to three years. Doubtless, in the near future, there will be a readiness for further attainments in this respect. The reports made upon the schools, gave the number in attendance at thirty-five colleges, during the last term at 5,532, and the graduates for the same time 1,363. The figures elicited a good deal of interest and some anxiety, on the part of a few at least, lest the profession in the near future should become crowded. Certainly there is no occasion for fear or anxiety in this direction up to the present time, and especially when we consider that quite a goodly per-cent of those who press themselves through the course have far from an adequate preparation for their professional duties, and will, many of them, at least, drop out of practice in a little while.

With a view of determining more fully than heretofore the true character and status of colleges applying for membership in this association, a special committee was appointed whose duty it shall be to visit any school applying for membership, during its session, personally examine its facilities for teaching, methods of instruction and efficiency of the faculty, and report to the Executive Committee, which report shall, if favorable, be acted upon.

This action, if well carried out, will be of great value to the Association, and enable it to act more intelligently than has been possible under the former method; it will also serve as a stimulus to all colleges seeking membership in the body.

With a view to purifying the educational atmosphere, the following action was had:

"WHEREAS, In view of the various reports being circulated derogatory to the character of certain schools without any one being willing to prefer charges sustaining such statements;

"*Resolved*, That the Executive Committee be, and is hereby authorized to exercise full power to investigate all such innuendoes or charges, by visiting the school or schools, or authorize some one to perform this duty, summoning witnesses, etc., in order that all such statements shall be sustained or proven false."

This action will have a tendency to greater caution in regard to rash and, often unfounded, statements that are indulged too often, and that without any real grounds; it will also, in some degree at least, have a wholesome influence upon the colleges.

Upon the whole, we regard this as one of the most profitable meetings this body has ever held. It is to be hoped that each year may see an equal or greater advance.

